# Predicting dive start performance from kinematic variables at water entry in (sub-)elite swimmers

**DOI:** 10.1371/journal.pone.0241345

**Published:** 2020-10-30

**Authors:** Marit P. van Dijk, Peter J. Beek, A. J. Knoek van Soest

**Affiliations:** 1 Department of Human Movement Sciences, Faculty of Behavioural and Movement Sciences, Amsterdam Movement Sciences, Vrije Universiteit Amsterdam, Amsterdam, The Netherlands; 2 Department of Biomechanical Engineering, Faculty of Mechanical, Maritime and Materials Engineering, Delft University of Technology, Delft, The Netherlands; São Paulo State University (UNESP), BRAZIL

## Abstract

The dive start is an important component of competitive swimming, especially at shorter race distances. Previous research has suggested that start performance depends on kinematic variables pertaining to the swimmer at water entry, notably the distance from the block, the horizontal velocity of the centre of mass and the angle between body and water surface. However, the combined and relative contributions of these variables to start performance remain to be determined. The aim of the present study was therefore to develop a model to predict start performance (time from take-off to reaching the 15-m line) from a set of kinematic variables that collectively define the swimmer’s entry state. To obtain an appropriate database for this purpose, fifteen well-trained, (sub-)elite swimmers performed dive starts under different instructions intended to induce substantial variation in entry state. Kinematic data were extracted from video recordings of these starts, optimised and analysed statistically. A mixed effects analysis of the relation between entry state and start performance was conducted, which revealed a significant and robust dependence of start performance on entry state (χ2(3) = 88, *p* < .001), explaining 86.1% of the variance. Start time was reduced by 0.6 s (*p* < .001) when the horizontal displacement at water entry was 1 m further, by 0.3 s (*p* < .001) when the horizontal velocity of the centre of mass was 1 m/s higher, and by 0.5 s (*p* < .01) when the entry angle was 1 radian flatter. The robustness of the analysis was confirmed by a similar mixed effects analysis of the relation between entry state and time to the 5-m line. In conclusion, dive start performance can be predicted to a considerable extent from the swimmer’s state at water entry. The implications of those findings for studying and improving block phase kinetics are discussed.

## Introduction

Most competitive swimming events, with the exception of backstroke and medley relay races, begin with a dive start. Especially at shorter race distances, the quality of the dive start contributes considerably to the overall race result. This is evidenced by the fact that the start performance is strongly related to overall race time for 50-m, 100-m and 200-m race events [1–3]. The dive start is typically defined to begin with the start signal and end with the swimmer’s head passing the 15-m line [3]. It can be subdivided in four phases: the block phase (i.e. from start signal to take-off from the starting block), the flight phase (from take-off to water contact), the underwater phase (from water contact to the swimmer’s resurfacing, the so-called ‘break-out’), and the start of free swimming (from the break-out to the 15-m line, which, according to the regulations of the Fédération Internationale de Natation (FINA), marks the point at which the underwater phase has to be completed). Each of these phases requires specific skills that are essential for a competitive dive start. For instance, during the block phase it is critical to assume a proper starting posture, and to generate explosive, properly coordinated push-off forces by both legs, depending on the start technique used [4]. During the flight phase, the swimmer’s body segments need to be properly aligned with the velocity vector of the centre of mass to allow a smooth entry into the water with a small frontal area so as to minimise water resistance [5,6]. The same holds for the underwater phase, which consists of a glide phase and a propulsion phase, involving undulation without arm and leg movements [7].

Each of the distinguished phases and associated actions of the dive start can be and have been studied in isolation [8–14], but one has to realize that the actions performed during one phase determine the initial conditions and thus the appropriate actions in the next phase. Therefore, the methodological question that arises is how start performance should be analysed in order to better understand both the organization of each phase and the causal chain of dependencies from one phase to the next. A common approach is to assess start performance in terms of the time to the 5-m, 7.5-m or 10-m line and to correlate selected performance related variables to those times [10,15–19]. Although useful insights into relevant performance determining variables may be obtained in this manner, these distances, and the associated times, do not correspond to the aforementioned start phases. Since swimmers enter the water well before crossing the 5-m line [20,21], the time required to do so is also dependent on the characteristics of the underwater phase [22], and the same holds for the 7.5-m and 10-m line. As a result, using this approach, it is difficult to gain a clear understanding of how the actions in one phase affect the actions in the next, and how the overall start performance results from the performance in each phase. To gain a better understanding of the dive start in swimming and its key performance-related variables, it seems therefore more expedient to examine the aforementioned phases in a piecemeal fashion and to determine the optimal (kinematic) state of the swimmer at the end of each phase rather than to correlate potential performance-related variables with arbitrarily defined distances and times. A suitable starting point in this regard seems to be an analysis of the kinematic variables associated with the swimmer at water entry, which collectively define the swimmer’s entry state. There are both theoretical and empirical reasons for this.

Theoretically, the position and velocity of the centre of mass (COM) at water entry are completely determined by the swimmer’s (kinematic) state at the end of the block phase if air resistance is neglected, while the entry state is by definition independent of the underwater phase [9,16,23]. Moreover, the entry state determines the initial part of the underwater phase, during which the velocity of the swimmer is drastically reduced due to water resistance [11]. From water entry onward the swimmer continues to glide underwater at a decreasing velocity and along a certain trajectory [11], which in turn determines the subsequent phase of active propulsion using dolphin kicks. The entire underwater performance, which strongly contributes to overall start performance [21,24–26], is hence dependent on the swimmer’s state at water entry. Adopting a focus on entry state, as done here, may thus provide insight into the (optimal) outcome of the block phase and the (optimal) initial conditions for the subsequent underwater phase.

There are also strong empirical grounds for adopting a focus on entry state to better understand the dive start. Previous research has identified several kinematic variables pertaining to the swimmer’s state at water entry that are associated with dive start performance, including the swimmer’s distance from the block [10,20,21,27], the horizontal velocity of the swimmer’s centre of mass [9,10,24], and the swimmer’s angle with the water surface [21]. That these variables contribute to start performance can be readily understood from basic mechanical considerations. A longer horizontal flight phase is beneficial because water resistance is much larger than air resistance, resulting in a rapid decrease of the swimmer’s velocity from water entry onward [11]. A greater horizontal velocity at water entry is beneficial because a successful start requires that a fixed horizontal distance, involving an aerial and an aquatic phase, is traversed in as short a time as possible. Finally, the entry angle is an important factor because it determines the frontal area of the swimmer and thus the water resistance at and after entry, as well as the shape of the underwater trajectory.

Even though these previous results indicate which variables at water entry are relevant to consider, there are two other regards in which the study of the dive start in swimming might be improved relative to the existing literature besides adopting a focus on entry state. Firstly, it is not only important to determine which (potential) performance-related variables contribute to start performance, but also their relative contribution compared to other variables. This requires not only that multiple rather than simple regression techniques are employed, as has been done in several previous studies (e.g., [20,24], but also that the analysis is applied to a data set with sufficient variation in the variables of interest, such that meaningful statistical patterns can be reliably detected and quantified. To achieve this, larger than usual variations in the swim starts need to be induced experimentally. Secondly, to deepen the understanding of the relation between (potential) performance related variables and start performance, and to determine their relative contributions to overall start performance, an explicit model needs to be developed and validated on the basis of empirical data. To our knowledge, however, no such modeling attempts have been made in previous studies on the dive start.

In the present study, we took all of the aforementioned considerations into account with the aim to determine the relation between entry state and start performance, as well as the relative contribution of the kinematic variables at water entry to the time from take-off to the 15-m line. In particular, we aimed to test three hypotheses that were based on a combination of the previous findings and theoretical considerations highlighted in the preceding: 1. Entry state is a strong predictor of start performance and accounts for a considerable amount of variance in overall start time; 2. The entry distance (i.e. the distance from the COM position from the block at water entry), the horizontal velocity of the COM at water entry, and the angle between the swimmer’s body and the water surface contribute to the relation between entry state and start performance; and 3. Entry distance and horizontal velocity of the COM are the strongest contributors to this relation, followed by entry angle. To test these hypotheses, we developed a model for predicting this performance measure based on data derived from an intentionally broad variation of front crawl dive starts performed by well-trained, (sub-)elite swimmers. To evaluate the robustness of this model, and to gain insight into the relative contributions of the entry state variables and how these evolve over the underwater phase, we derived a similar model for the relation between entry state and time to the 5-m line.

## Materials and methods

### Participants

A similar number of participants were invited to participate as in previous studies on the dive start (8 participants in [8], 13 in [18], and 4 in [22] respectively), but with the intention to have them perform more, and more varied, start dives than in those studies. Three elite and twelve sub-elite swimmers with at least three years of experience in competitive swimming at a national level were recruited for this purpose. Participants were included when they were 14 years of age or older and when their start performance (i.e. time elapsed between start signal and crossing the 15-m line) was considered good to very good by their coach. All participants trained between 12 and 20 hours per week on average, and had qualified for the Dutch national championships of 2018 in their respective age groups. Their performance level was quantified in terms of FINA points. The FINA points were calculated based on each participant’s best time in his/her main event, which was scaled down from 1000 points based on the global 2020 fastest senior performance in this event. The points ranged from 601 to 741, with a group average of 660, see Table 1. The main events of the participants were 50m (N = 7), 100m (N = 1), 200m (N = 5) and 800m (N = 1) freestyle or butterfly. In addition, the group average of the best start times achieved by the participants during the experiment is reported in Table 1, which ranged from 6.42 to 8.22 s. The participants’ best start times were similar to those reported in previous studies on the swimming start with (Slovenian) national swimmers [10] (10) and the elite participants’ start times were similar to those reported in studies with elite swimmers [28]. The participants, or their parents or legal guardians in case they were younger than 16 years of age, were informed about the procedures and provided informed consent before the experiment. The study was approved by the ethics committee of the Faculty of Behavioural and Movement Sciences, Vrije Universiteit Amsterdam, and conducted in full compliance with the Declaration of Helsinki.

**Table 1:** Characteristics of included participants (mean ± standard deviation) as acquired by means of anthropometrical measurements (mass and height) and personal communication with the trainer (age).

	Men (N = 8)	Women (N = 6)
Age (years)	18 ± 1	17 ± 2
Height (m)	1.86 ± 0.04	1.73 ± 0.04
Mass (kg)	78.9 ± 5.0	65.6 ± 7.5
FINA points	660 ± 45	686 ± 38
Best start time (freestyle) to the 15-m line (s)	6.91 ± 0.32	7.81 ± 0.35

### Procedure

The experiment was conducted in the InnoSportLab de Tongelreep in Eindhoven, a field lab for elite swimming equipped with multiple under- and above-water digital video cameras, a custom-made system for measuring active drag, a time registration system, a force-instrumented starting block and turning point, and various wearable sensor systems. The participants visited this facility on three occasions (see Table 2) so as to minimise the influence of fatigue on performance; they were asked to arrive in the facility in a ‘rested’ state. Before each session, the participants were instructed to perform freely selected warm-up exercises on land (allowing them to use their own warm-up routines) for a duration of ten minutes followed by a series of standardised squat jumps to verify that their ‘freshness’ or ‘performance readiness’ (in terms of neuromuscular fatigue) was relatively constant across test sessions. Subsequently, they performed eight freestyle dive starts in different conditions (see below), which were recorded on video. All test sessions were conducted at the same time of day and within a time span of eight days, thus avoiding substantial differences in ‘freshness’ between sessions. The test sessions were held early in the morning, such that no intense training sessions were performed in the 12 hours preceding the trials.

**Table 2:** Example of an individual protocol for the three test sessions (S1–S3 Tables). Fig 1 provides a visual representation of all six conditions and a more elaborate explanation.

Session	Squat jump test	Starts in different conditions			
S1	3x Squat jump	2x Without arms	2x Regular	2x Without arms	2x Regular
S2	3x Squat jump	2x Steep take-off	2x Short BT*	2x Steep take-off	2x Short BT*
S3	3x Squat jump	2x Flat take-off	2x Submax. effort	2x Flat take-off	2x Submax. Effort

* BT = block time.

**Fig 1:**
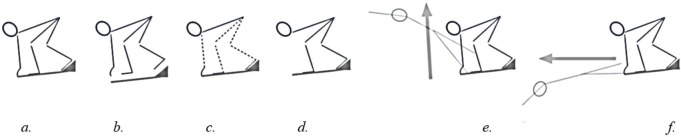
Visual representation and explanation of the six different start conditions. The start conditions were labelled as (a) ‘regular’, (b) ‘block time as short as possible’, where block time was defined as the ‘time from the start signal until take-off (i.e. the last instant at which the swimmer is in contact with the block)’, (c) ‘block phase at submaximal effort’, i.e. participants were instructed to perform maximally only from the moment they entered the water, (d) ‘block phase without using arms’, (e) steep take-off, and (f) flat take-off. Since the take-off angle was defined as the angle between the line from toe to hip and the horizontal at take-off, a steep take-off and a flat take-off correspond to a large and small (positive) take-off angle, respectively.

In total, there were six different start conditions (Fig 1), five of which were atypical and implemented by instruction. The conditions were designed to induce sufficient variation in water entry to detect meaningful statistical patterns. The instructions in question (see S1 Appendix) were given with regard to the block phase rather than water entry to preclude the swimmer from making consciously controlled adjustments during the entry and underwater phase. Because all instructions contained an element of subjective interpretation and a learning effect could occur over time in executing them, a certain degree of variability within conditions was inevitable. However, this variability within conditions was not considered problematic as it added to the aim of inducing variation in water entry, besides the deliberately induced variation between conditions.

Instructions and conditions were tested in a pilot study, which was conducted one month before the start of the experiment. In this pilot study two swimmers, with a performance level similar to the participants of the experiment proper, ran through all sessions of the experimental protocol to determine if the instructions had the desired effect and if there were any unintentional side effects. After the sessions, the instructions were discussed with the swimmers and adapted on the basis of their feedback if deemed appropriate. In addition, all starts were analysed to verify model assumptions and assessment criteria. No adaptations in the experimental protocol were made as a result of the pilot study.

On each measurement day, participants performed two of the six conditions. Each condition was repeated four times, resulting in eight starts per session. The condition ‘regular’ was always performed during the first session, and served as baseline to determine thresholds, in terms of take-off angle and block time, for the other conditions (see S1 Appendix). The order of the other conditions was randomised for each participant over and within the subsequent two measurements days. An example of an individual test protocol is shown in Table 2. Participants were instructed to perform all starts at maximal effort (unless instructed otherwise) including the underwater phase, breakout and subsequent front crawl swimming until the head reached a distance of 18m, which was indicated with a marker at the bottom of the pool. Between the starts, participants rested for three minutes. To minimise interference between conditions, two consecutive starts were performed per condition. Directly after execution it was evaluated whether the start was appropriate according to a set of preconceived assessment criteria (see S1 Appendix). If a start did not meet all of those criteria, the trial in question was discarded and repeated immediately. If the swimmer’s arms, legs or trunk were not fully extended at water entry, the starts were excluded from further analysis. The start signal was generated automatically with a random duration between ‘take your marks’ and the start signal in order to mimic race conditions. Definitions and calculations of the predictor and response variables of interest are provided in Table 3.

**Table 3:** Definitions of the predictor (independent) and performance (dependent) variables.

Variable	Description
Predictor variables	
Position COM at water entry (m)	The horizontal and vertical position of the centre of mass (COM) at water entry.
Position hand at water entry (m)	The horizontal and vertical position of the fingertips. This variable is will be referred to as entry distance.
V_x_ of the COM at water entry (m·s^-1^)	The horizontal component of the velocity vector of the COM at water entry.
V_y_ of the COM at water entry (m·s^-1^)	The vertical component of the velocity vector of the COM at water entry.
V direction at water entry (rad)	The angle between the direction of the velocity vector of the COM and the horizontal at water entry.
V magnitude at water entry (m·s^-1^)	The magnitude of the velocity vector of the COM at water entry.
Entry angle at water entry (rad)	The angle between the trunk and the horizontal at water entry. For all angles, a downward direction from the horizontal was defined as negative.
Position COM at COMcrossWL (m)	The horizontal and vertical position of the COM at the moment the COM crosses the waterline (i.e. COMcrossWL).
V direction at COMcrossWL (rad)	The angle between the direction of the velocity vector of the COM and the horizontal at COMcrossWL.
Entry angle at COMcrossWL (rad)	The angle between the trunk and the horizontal at COMcrossWL.
Direction difference at COMcrossWL (rad)	The absolute angle between the direction of the COM velocity vector and the trunk at COMcrossWL.
Response variables	
Time to the 15-m line (s)	Time from the start signal until the head passes the 15-m line
Time from take-off to the 5-m line (s)	Time from take-off until the hip passes the 5-m line (i.e. TTO5)
Time from take-off to the 15-m line (s)	Time from take-off until the head passes 15 m (i.e. TTO15)
*z*-score15m	Standardised time to the 15-m line (with mean = 0 and standard deviation = 1) for each swimmer separately.
Horizontal hip velocity at 5 m (m.s^-1^)	Average horizontal velocity of the hip at 5 m distance from the wall which was calculated by dx/dt of the hip marker between the instant the hip marker crosses 4.5 m and the instant the marker crosses 5.5 m.
Outcome5m	Performance measure at 5 m including both time and horizontal hip velocity at 5 m calculated by: horizontal hip velocity at 5 m * (1/TTO5)

All kinematic variables are relative to a frame of reference with origin located at the intersection between the water surface, the wall at the beginning of the pool in the plane of movement, and half a body width (0.15 m) away from the centre of the starting lane.

To detect potential differences in the participants’ freshness between test sessions, the squat jump height was determined at the start of each session. This is considered a representative freshness measure for dive start performance because, like the dive start, the squat jump requires a quick build-up of muscle stimulation and force [29,30] without making a countermovement. Squat jumps were initiated at a knee angle of 90 degrees; participants were instructed to keep the upper body as upright as possible and to place their hands on their hips to prevent them from making a contribution to the jump. This position was held for 2 s after which the participant was encouraged to jump as high as possible. Three jumps were performed with a 1-minute rest interval in between. The average of the highest two jumps was used in the analysis.

Body segment inertia parameter values of the participants were determined using anthropometric measurements. Length and circumference of the forearms, upper arms, head + neck, thorax, abdomen, pelvis, thighs, shanks and feet were measured in accordance with the method described by Zatsiorsky [31]. These measures were then used to estimate mass, COM position and moments of inertia relative to the COM of all segments. In addition, total body mass was measured to calculate the whole-body COM position and to check mass estimations of the segments. All anthropometric measurements were conducted by the same qualified investigator.

### Equipment and experimental set-up

All starts were performed from a starting block with the same dimensions as the Omega OSB11 starting block as currently used in all major international swimming competitions. The swim starts were recorded in the sagittal plane by five digital video cameras (Basler, Germany). One camera was positioned above water on one of the lateral walls of the swimming facility (3.6 m from the centre of the swimming lane), perpendicular to the starting block, while the other four cameras were positioned under water in the lateral wall of the swimming pool, perpendicular to the 2.5-m, 5-m, 10-m and 15-m point, respectively. The sampling frequency was 50 Hz and the video images had a resolution of 788×524 pixels. Although it has been suggested that a sampling frequency of 100 frames per second is needed for a reliable full biomechanical analysis of the dive starts in swimming [32], a sampling frequency of 50 Hz was used in the present study, as in previous studies [13,16,20]. This sampling frequency was considered appropriate for the purpose of the present biomechanical analysis, because the dependent variables in this study did not require calculation of second order derivatives. The video images were synchronised with the start signal by flashing a light at the time of the start signal and captured using Streampix software (Norpix, Streampix 7, 2016).

Kinematic analyses were performed on the calibrated video frames using customised StartAnalyzer software, running on a PC (Escrito sport, Eindhoven, The Netherlands). Video frames were calibrated by placing a screen with known dimensions and positions relative to the origin in the plane of motion (3.6 m from the lateral wall). Based on this calibration, each pixel in the video frames was translated to real-world coordinates within this plane. The origin was defined as the intersection between the water surface, the wall at the beginning of the pool in the plane of motion, and half a body width (0.15 m) from the centre of the starting lane. It was verified visually that swimmers remained in this centre.

Jump height was measured using a vertical jump meter (JUMP-MD, T.K.K. 5406), consisting of a measurement cord that was connected to a belt around the participant’s waist and a mat on the ground. Jump height was defined as the maximal difference between the height of the participants’ belt when standing upright with the heels on the ground and the height during the jump as determined with the measurement cord. A goniometer was used to indicate the right knee angle in squat position. Anthropometric measures were determined with an anthropometric measurement set with a standardised measurement protocol.

### Data analysis

#### Entry state model

To examine the relation between entry state and start performance, a dynamic entry state model was built. Firstly, for each start, the kinematic variables defining the swimmer’s entry state were obtained through non-automated video analysis. Subsequently, these variables were optimised to render them mechanically consistent with model assumptions and known external forces. Finally, the evolution of the entry state variables over time was simulated using a forward dynamic model. These steps are explained in more detail in this sub-section and the next.

Based on pilot data and exclusion criteria, it was assumed that the swimmer’s arms, legs and trunk were fully extended (but not necessarily aligned) when entering the water, that both arms were in the same sagittal plane, and that the head was not necessarily aligned with the trunk. The dynamic model that emanated from these considerations consisted of five rigid segments (left leg, right leg, trunk, combined arms, and head), which were connected at the hip and shoulder, respectively. To obtain the kinematic variables of interest for each start, the positions of the ankles (most lateral and medial points of malleoli), hip (trochanter major), shoulder (acromion), cranial vertex and wrist (stylion) were analysed in the sagittal plane at water entry as well as one frame (0.02 s) before and one frame after water entry. Except for the acromion and trochanter major, to which a marker was attached, the positions of the other anatomical landmarks were determined visually.

The reliability of the non-automated video analysis was determined. Based on 40 random starts of four participants the test-retest reliability in terms of the mean absolute difference between two analyses of the same start was 0.7 ± 0.8 cm. To further reduce noise and to obtain a description of the kinematics that was consistent with the rigid-body assumptions, the constrained optimization algorithm proposed by Faber et al. [33] was used. This resulted in slightly adapted kinematic data that were mechanically consistent with the known external forces while deviations from the actually measured positions were minimised. The adapted kinematic data were used to calculate whole body COM position and velocity at water entry and at the instant the COM crossed the waterline (COMcrossWL). Subsequently, the direction and magnitude of the COM velocity vector were decomposed in a vertical and a horizontal component, which were used for further analysis. In addition, the hand position at water entry (defining the entry distance), entry angle at water entry, entry angle at COMcrossWL, and the difference between entry angle and the direction of the COM velocity vector at COMcrossWL were calculated (see Table 3). Variables at COMcrossWL were obtained by extrapolating the entry state model, such that the development of variables over time was modelled. This will be explained in the next section.

#### Development of entry state model

Since some variables, such as entry angle and frontal area, were expected to influence start performance while crossing the water line, rather than at water entry, the positions and direction of the entry state model were extrapolated in order to obtain these variables at COMcrossWL. In this manner, the effect of variables at COMcrossWL on start performance could be assessed.

It was assumed that, during immersion, the whole-body angular momentum remained constant, the body was moving towards full extension, and the entry hole diameter, i.e. the size of the hole created by the swimmer when penetrating the water surface (measured by the distance between the extreme points of the swimmer in the sagittal plane) was minimised. Based on these assumptions, a forward dynamics simulation of the swimmer’s behaviour was performed, from water entry until all segments were completely immersed. Equations of motion were derived using the method described in Casius et al. [34]. The second assumption was realised in the model by imposing visco-elastic joint moments that ‘pull’ the joints to full extension. In particular, resting joint angle for each joint was set at full extension, joint rotational stiffness at 1000 Nm/rad and joint rotational damping at 150 Nms/rad. These values were determined heuristically, considering joint rotational stiffness values ranging from 600 to 1000 Nm/rad, resting joint angle values ranging from 0 to 20 degrees joint angle, and joint rotational damping values ranging from 5 to 150 Nms/rad. We selected the combination of parameter values that resulted in the smallest averaged entry hole. (Note: since segments in the chain model are represented by lines, entry hole diameter is based on segments without cross-sectional area.) In this way, body segments approached alignment when the body immersed. Subsequently, the COM velocity vector and entry angle at COMcrossWL predicted by this model were calculated.

#### Assessing start performance

Start performance was assessed as the time from take-off to the 15-m line (TTO15), that is, without taking the block time into account. This was necessary because block time could be affected by the instructions imposed to induce variation in the dive starts. To examine whether results of the statistical analysis are robust for each (deliberately) chosen distance, a second entry state model was created with time from take-off to the 5-m line (TTO5) as response variable. Comparison of these two entry state models may give insight into the relative contributions of the entry state variables and how these evolve over the underwater phase, i.e. from the 5-m to the 15-m line.

### Statistical analysis

Before the relation between TTO15 and entry state could be determined, a selection of the most important entry state variables was made. To prevent a bias in this selection caused by style differences between skilled and less skilled starters or by inter-individual performance differences of the underwater phase, two additional response measures (*z*-score15m and outcome5m) were determined to make this selection. The first measure, *z*-score15m, was calculated to standardise TTO15 for each swimmer by subtracting the mean and dividing the resulting difference by the standard deviation of TTO15 of all starts performed by the swimmer in question. This measure was used to exclude any bias as a result of start proficiency. The second measure, outcome5m, quantifies the swimmer’s performance up to the 5-m line. This measure was used to exclude possible influences of the underwater trajectory. Since both time and velocity at 5 m will affect the performance at the 15-m line (e.g., the best start time is likely to be achieved when the time at the 5-m line is low and the horizontal velocity at 5 m is high), outcome5m combines those measures. The variable represents a score composed of both the horizontal hip velocity at 5-m and the time from take-off to the 5-m line (TTO5) and was calculated by horizontal hip velocity at 5 m * (1/TTO5). A high outcome5m-score thus corresponds to a good performance of the first 5 m.

The initial selection of entry state variables was made based on results of three simple regression analyses (with entry variable as explanatory variable and, respectively, TTO15, z-score15m and outcome5m as response variable) and pairwise Pearson correlations. Firstly, variable selection was based on the three *p*-values from the simple linear regression. If one or more of these *p*-values was larger than 0.3, the variable was excluded. Subsequently, from the remaining variables that showed an absolute collinearity of 0.75 or higher, the one with the highest average bearing on the simple linear regression was selected.

To investigate the relation between start performance and variables at water entry, a linear mixed effects analysis was performed [35]. The explanatory variables of the mixed model consisted of the initial selection of entry variables, and the response variable was TTO15. To arrive at a concise model while avoiding the risk of over-fitting, a step-down method was used. With this method, one starts with the ‘full model’ including all explanatory variables and then removes the explanatory variable *j* that has the highest *p*-value for H0: β_j_ = 0, until all explanatory variables in the model are significant. The remaining variables were included as fixed effects in the model. Since the swimmers in this study represent a sample from a population of swimmers, their effects were considered as a sample from a population of effects. Therefore, individual effects were modelled as random effects within the model. Finally, elaboration of the model with gender was assessed. If adding this variable led to a change of ≥ 10% of the model coefficients, it was identified as a confounder and added to the model.

After the model was derived, it was validated and evaluated. To assess the validity of the model, its assumptions were tested. Firstly, the linearity of the relations was assessed by visually inspecting the partial residuals. If these plots indicated that the relations deviated from linear, linearization of the predictor variables or response variable was investigated. Secondly, the model was tested for multi-collinearity by calculating the variance inflation factor. This factor makes use of R-squared values of regressing each predictor against all other predictors and can be used to check for indirect correlations between the predictor variables. The variance inflation factor ranges from 1 upward, where a value of 1 indicates no correlations and >5 indicates that explanatory variables are highly correlated. Thirdly, when using a mixed effects regression, it is assumed that random effects came from a normal distribution. This assumption was checked by performing a Shapiro-Wilk normality test on the random effects of the model (i.e. the random effect of the variable ‘participant’). The last assumption to be checked was a reasonable spread of the residuals along the fitted values and the absence of unexplained patterns when the fitted values are plotted as a function of the input variables. Therefore, the predicted values for TTO15 were plotted against the measured values and a visual check was performed.

After validation, the goodness-of-fit of the model was determined by calculating the likelihood ratio statistic of the comparison of the full model (including all initially selected predictor variables) and the final model. In addition, the variance explained by the final model and the mean difference between the calculated TTO15 and the TTO15 predicted by the model were calculated. The final step was to confirm statistically whether entry state variables affect start performance. This was done by comparing the likelihood ratio statistic of the final model with that of the model without entry state variables. A significant difference in this regard would confirm the influence of entry state on start performance. The difference in standard deviation of the residual between those two models was also reported. An identical procedure (from mixed effects analysis until validation and confirmation) was performed for TTO5 as response variable.

Finally, to verify the assumption that the participants’ freshness would be similar over sessions, a one-way ANOVA with session as within-participant factor was performed and repeated measures on squat jump heights was performed.

All statistical analyses were conducted in software R [36], using the packages ‘matrix’ [37], ‘lme4’ [38], ‘MuMIn’ [39] and ‘car’ [40]. The significance level was set at *p* < 0.05.

## Results

Of the 15 participants included in the study, one female participant was excluded due to camera failure during one of the test sessions. Logistic reasons precluded repeating this session. Four starts were excluded because the legs were not fully extended at water entry. The general characteristics of the 349 starts that were included in the analysis are listed in Table 4.

**Table 4:** Overview of the values (mean ± standard deviation) for the variables of all starts (left) and for the variables of the ‘regular’ starts (right) for men (N = 8) and women (N = 6).

	Based on all starts		Based on ‘regular’ starts	
Variable	Men	Women	Men	Women
Time to the 15-m line (s)	7.40 ± 0.56	8.16 ± 0.44	7.03 ± 0.32	8.03 ± 0.43
TTO15 (s)	6.61 ± 0.56	7.34 ± 0.40	6.30 ± 0.34	7.25 ± 0.40
Horizontal position COM at water entry (m)	2.1 ± 0.3	1.9 ± 0.2	2.2 ± 0.3	2.0 ± 0.1
Horizontal position hand at water entry (m)	3.2 ± 0.3	2.8 ± 0.2	3.3 ± 0.3	2.8 ± 0.1
V_x_ of the COM at water entry (m/s)	4.4 ± 0.3	4.0 ± 0.3	4.6 ± 0.2	4.1 ± 0.3
V_y_ of the COM at water entry (m/s)	3.1 ± 0.3	3.2 ± 0.3	3.2 ± 0.3	3.2 ± 0.3
V direction at water entry (rad)	-0.61 ± 0.06	-0.67 ± 0.07	-0.61 ± 0.05	-0.66 ± 0.07
V magnitude at water entry (m/s)	5.4 ± 0.3	5.1 ± 0.2	5.6 ± 0.2	5.2 ± 0.2
Entry angle at water entry (rad)	-0.39 ± 0.12	-0.35 ± 0.10	-0.37 ± 0.08	-0.33 ± 0.00
Horizontal position COM at COMcrossWL (m)	3.0 ± 0.3	2.6 ± 0.2	3.0 ± 0.3	2.7 ± 0.1
V direction at COMcrossWL (rad)	-0.85 ± 0.05	-0.89 ± 0.05	-0.84 ± 0.03	-0.87 ± 0.04
Entry angle at COMcrossWL (rad)	-0.67 ± 0.11	-0.74 ± 0.12	-0.62 ± 0.13	-0.71 ± 0.10
Direction difference at COMcrossWL (rad)	0.18 ± 0.10	0.16 ± 0.11	0.22 ± 0.12	0.18 ± 0.11

### Impact of conditions

During the sessions, participants performed starts under different instructions, which induced substantial variation in entry state, as intended. Table 5 shows the values of the block and entry state variables for each condition. Although block time was on average 0.05 s shorter in the ‘short block time’ condition, the values of the different entry state variables for this condition did not deviate much from those for the regular starts. In contrast, conditions with deviating take-off angles led to substantiable differences in horizontal velocity at water entry, entry distance and entry angle. The condition in which block phase was performed at submaximal effort led to lower COM horizontal velocities and a shorter distance at water entry. The ‘without arms’ condition resulted in longer block times and lower COM horizontal velocity at water entry only.

**Table 5:** TTO15 and block and entry state variable values for each condition (mean ± standard deviation).

	TTO15 (s)	Block time (s)	Take-off angle (rad)	V_x,COM_ (m/s)	Entry distance (m)	Entry angle _COMcrossWL_ (rad)
Regular	6.71 ± 0.60	0.75 ± 0.05	0.47 ± 0.13	4.4 ± 0.3	3.1 ± 0.3	-0.66 ± 0.12
Short block time	6.78 ± 0.53	0.70 ± 0.05	0.47 ± 0.14	4.4 ± 0.3	3.1 ± 0.3	-0.66 ± 0.11
Steep take-off	7.08 ± 0.59	0.82 ± 0.09	0.66 ± 0.17	3.9 ± 0.3	3.2 ± 0.3	-0.76 ± 0.12
Flat take-off	7.12 ± 0.72	0.82 ± 0.06	0.35 ± 0.10	4.3 ± 0.4	2.8 ± 0.3	-0.72 ± 0.11
Submax effort	6.98 ± 0.56	0.86 ± 0.07	0.48 ± 0.13	4.2 ± 0.3	3.0 ± 0.3	-0.72 ± 0.10
Without arms	6.86 ± 0.57	0.88 ± 0.08	0.48 ± 0.13	4.3 ± 0.3	3.0 ± 0.3	-0.70 ± 0.10
Total	6.92 ± 0.62	0.80 ± 0.09	0.48 ± 0.16	4.3 ± 0.4	3.0 ± 0.3	-0.70 ± 0.12

### Relation between start performance and entry state

The initial selection of water entry variables included in the full model and the variables included in the final model are reported in Table 6, which also shows the results of the simple linear regression analyses. The linear mixed effects analysis to describe the relation between start performance (TTO15) and water entry variables resulted in the following equation:

$$TTO15=9.623-0.310\;v_{x,COM,ES}-0.575\;X_{ES}-0.511\;{EA}_{COMcrossWL}$$ (1)

**Table 6:** Results of the simple linear regression analysis (initially selected^★^, included in the final model^■^). Results are presented as *r*^2^.

Variable	outcome5m	TTO15	*z*-score15m
Horizontal position COM at water entry	0.16 (*p* < .001)	0.04 (*p* < .001)	0.02 (*p* < .05)
Horizontal position hand at water entry^★■^	0.40 (*p* < .001)	0.19 (*p* < .001)	0.03 (*p* < .01)
V_x_ of COM at water entry^★■^	0.41 (*p* < .001)	0.32 (*p* < .001)	0.06 (*p* < .001)
V_y_ of COM at water entry^★^	0.01 (*p* < 0.05)	0.03 (*p* < .01)	0.02 (*p* < .01)
V direction at water entry	0.21 (*p* < .001)	0.20 (*p* < .001)	0.00 (*p* < .54)
V magnitude at water entry^★^	0.30 (*p* < .001)	0.19 (*p* < .001)	0.10 (*p* < .001)
Entry angle at water entry	0.00 (*p* = .96)	0.01 (*p* = .20)	0.03 (*p* < .01)
Horizontal position COM at COMcrossWL	0.39 (*p* < .001)	0.16 (*p* < .001)	0.01 (*p* = .04)
V direction at COMcrossWL	0.21 (*p* < .001)	0.19 (*p* < .001)	0.03 (*p* = .001)
Entry angle at COMcrossWL^★■^	0.11 (*p* < .001)	0.08 (*p* < .001)	0.08 (*p* < .001)
Direction difference at COMcrossWL	0.01 (*p* = .10)	0.00 (*p* = .37)	0.01 (*p* = .04)

The likelihood ratio statistic that was used for comparing the model without water entry variables with the final model (Eq 1) revealed that start performance was significantly affected by entry state (χ2 (3) = 98, *p* < 0.001). The standard deviation of the residual decreased from 0.26 to 0.23 s when entry state variables were added. According to the resulting model equation, TTO15 was reduced by -0.58 ± 0.08 s (*p* < 0.001, *r*^2^ = 0.19) for each m covered by entry distance (X_ES_), by -0.31 ± 0.06s (*p* < 0.001, *r*^2^ = 0.32) for each m/s in COM horizontal velocity at water entry, and by -0.51 ± 0.16s (*p* = 0.001, *r*^2^ = 0.08) for each radian in entry angle (EA) at COMcrossWL. The variance of TTO15 was 0.38, of which 85.5% was explained by the model, with a near-zero difference between the mean predicted TTO15 and the mean measured TTO15. The likelihood ratio statistic revealed small but non-significant differences in the goodness-of-fit between the full model and the final model (χ^2^(3) = 5.3, *p* < 0.15).

**Fig 2:**
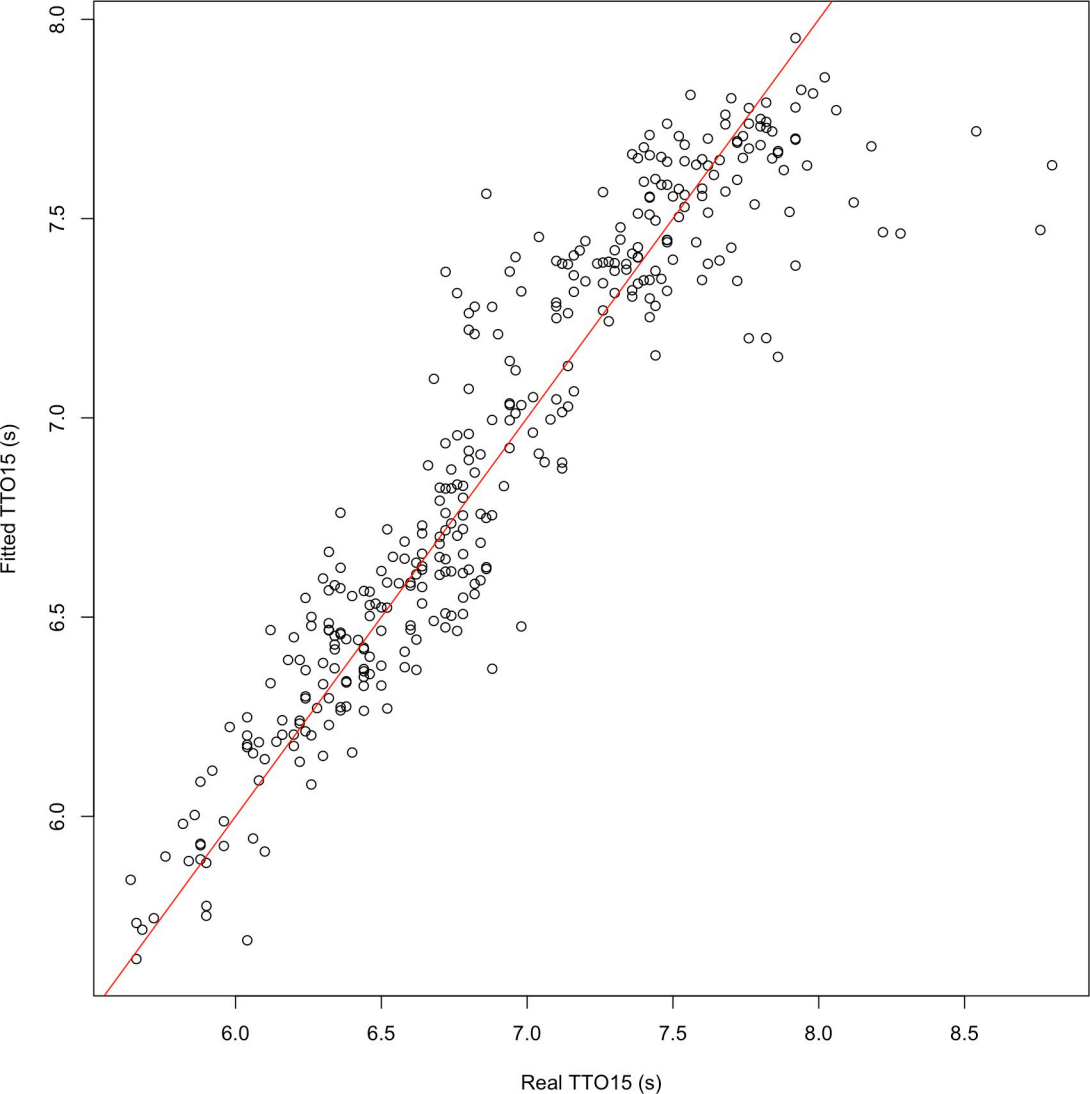
Plot of TTO15 predicted by the model against the measured TTO15 for all starts considered in the analysis (identity line indicated by red line).

Statistical checks of the model’s assumptions revealed linear partial relations and showed variance inflation factors of 1.11 for entry distance, 1.21 for COM horizontal velocity, and 1.22 for entry angle at COMcrossWL. Hence, no collinearity was observed. The Shapiro-Wilk normality test revealed a normal distribution of the random effects (W = 0.94, *p* = 0.43). Fig 2 shows a plot of the predicted TTO15 against the measured (or real) TTO15. No unexplained patterns were observed. The residuals were relatively equally distributed except for the higher (> 7.3) TTO15 values. This may have been due to underachievement of some participants, perhaps as a result of fatigue or lack of focus. However, since the number of deviating data points was relatively small, and partial residuals plots looked linear, the model was assumed to be valid.

Similar results were found for the linear mixed effects analysis when TTO5 was used as response variable. This model is presented in Eq 2 and revealed a significant effect of water entry variables on TTO5 (χ2 (3) = 267, *p* < 0.001). According to the resulting model equation, TTO5 was reduced by -0.11 ± 0.01 s (*p* < 0.001, *r*^2^ = 0.19) for each m covered by entry distance (X_ES_), by -0.14 ± 0.01s (*p* < 0.001, *r*^2^ = 0.32) for each m/s in COM horizontal velocity at water entry, and by -0.18 ± 0.03s (*p* = 0.001, *r*^2^ = 0.08) for each radian in entry angle (EA) at COMcrossWL. The variance in TTO5 was 0.02 of which 91.3% was explained by the model. Additional results and statistical checks are reported in S2 Appendix and indicate that this model is valid as well.

$$TTO5=2.033-0.144\;v_{x,COM,ES}-0.106\;X_{ES}-0.178\;{EA}_{COMcrossWL}$$ (2)

### Verifying participants’ freshness

The one-way repeated measures ANOVA that was performed to verify that the participants had a similar freshness across the three sessions revealed no significant effect of session on average squat jump height (*F*(1, 2) = 0.4, *p* = 0.7). The mean absolute differences (averaged over participants) were 1.5 cm between session 1 and 2, 1.5 cm between session 2 and 3, and 1.9 cm between session 1 and 3 (with an average of 46 ± 5 cm for the male participants and 39 ± 5 for the female participants).

## Discussion

Based on data gathered in an experiment in which variation in entry state was deliberately enhanced through instruction, we derived a model for predicting the relation between entry state and overall start performance in well-trained, (sub-)elite swimmers. Comparison with validation data indicated the model to be valid. The prediction model confirmed all our hypotheses. The first hypothesis was confirmed by the mixed effects analysis, which revealed that entry state is a strong predictor of start performance. The second hypothesis was confirmed by the fact that the three entry state variables that were included in the prediction model on statistical grounds, were the distance from the block at water entry, the horizontal velocity of the COM at water entry, and the entry angle at the instant the COM crossed the waterline. And the third hypothesis was confirmed by the finding that horizontal distance and velocity had the strongest association with start performance, and entry angle the weakest.

The three identified entry state variables are consistent with previous findings and conclusions regarding the main performance-determining variables of the dive start, which is an important replication result in its own right. Moreover, the start times (to the 15-m line), entry distances, horizontal velocities and entry angles for the regular starts of the current study were comparable in magnitude as those reported for high level swimmers in pertinent studies [10,13,20,21]. These studies used video analysis [13,20,21] and/or force plates to measure kinematic variables related to the swim start. In the following paragraphs, the three main entry state variables are discussed in further detail with respect to previous findings.

Entry distance also emerged as a strong contributor to start performance in the study by Peterson Silveira at al. [20], who examined the main biomechanical variables determining start time (5 m) in different start technique variants (grab start, rear- and front-weighted track and kick start) in breaststroke swimmers. Using a Lasso regression, they found that start time (s) was lowered by -0.35 s for every m of entry distance, as opposed to the -0.60 s/m found in our study. An explanation for this difference may be that the different start techniques studied by Peterson Silveira et al. [20] resulted in markedly less entry distance variability than brought about in the present study.

The importance of COM horizontal velocity at water entry was also established by Tor et al. [24], who investigated key variables of the start and their relation to the 15-m time in elite swimmers (among which 39 Olympians). Using a multiple regression analysis, the horizontal and vertical velocity of the COM at take-off emerged as the two main above-water variables related to start performance. They reported that the 15-m time (s) was lowered by -1.42 s for every m/s in take-off horizontal velocity, as opposed to the -0.30 s found in the present study. An explanation for this difference may be that Tor et al. [24] only included one start per swimmer for a total of 52 starts, compared to >24 starts per swimmer and a total of 349 starts in our study. As a result of individual differences in the execution of the swim starts [41], the inter-individual variation within the dataset of Tor et al. was probably large, while intrinsic technique differences between swimmers may have caused a bias towards the best starters included in the study.

A significant correlation between entry angle and start performance has been previously found by Ruschel et al. [21], albeit in a sample of four swimmers only and with little further analysis. In addition, Peterson Silveira et al. [20] found significant correlations between body angle at hip entry and time to the 5-m line. However, this angle was defined as the angle between the horizontal axis and the line through the hip and the ankle, and is different from the trunk angle as defined in our study. In the present study, we found a smaller (flatter) entry angle to be related to a better start performance, as did Peterson Silveira et al. [20]. This may be explained by the larger change in the direction of movement (or deflection) needed after entering the water at a steep angle. During deflection, the swimmer’s frontal area increases, causing an increased water resistance and hence a greater loss of velocity [14]. Although the relation between entry angle and start performance has been previously analysed in linear terms, this relation is probably nonlinear over the full range of achievable entry angles. A linear relation would mean that flatter is better, which is unlikely, since a horizontal trunk at water entry would result in a disadvantageously large frontal area. We therefore expect the relation between entry angle and TTO15 to be nonlinear, with a currently unknown optimum angle in the order of -35 degrees (based on the average entry angle of ‘regular starts’ in our study and [40]). Unfortunately, however, the current data set does not allow a detailed analysis of the precise nature of this relationship.

To conclude the comparison with pertinent literature, it is useful to highlight the added value of our study and the methodological aspects in which it differs from previous studies. Firstly, the present study stands out by focusing specifically on the importance of entry state for start performance, and in showing that a considerable amount of variance in start performance is accounted for by entry state alone. Secondly, where previous studies have identified which entry variables affect start performance on the basis of a limited number of starts and participants [20,24], the present study succeeded in determining the relative contribution of relevant kinematic variables to start performance by including a large number of starts per participant and by increasing the variation between starts by means of instruction. Both results were obtained by deriving an explicit prediction model for start performance on the basis of a set of kinematic entry state variables. The model was validated and thus provides a useful basis for future research on both the block phase kinetics and the underwater phase, as well as for deriving some practical guidelines for optimising the dive start and its training, as will be discussed next.

Since the entry state is largely determined by the swimmer’s state at take-off, a known desired entry state implies that the desired result of the block phase can be reconstructed. In other words, given a desired entry state, it is possible to reverse engineer the block technique resulting in that entry state. However, before doing so, it is necessary to first establish experimentally that manipulation of the identified entry variables affects start performance in accordance with the derived prediction model. Only when causality has been confirmed in this manner, which would imply that horizontal velocity at water entry and entry distance should be maximised, can the optimal block technique be reconstructed. While increasing the magnitude of the velocity vector may be limited by biomechanical and neuromuscular properties, the direction of this vector can be optimised. Note that COM horizontal velocity at water entry equals the horizontal component of the velocity vector at take-off if air resistance is neglected. Besides, entry distance can be calculated from the vertical and horizontal component of the velocity vector at take-off, gravitational acceleration, and the vertical distance between the COM position at take-off and the water surface. Because both the entry distance and the horizontal velocity of the COM at water entry depend on the direction of the velocity vector at take-off, the direction of this vector can be tuned such that the model value is maximised. Since the current study determined the importance hierarchy of the three entry state variables, the same prioritization of these variables should be imposed when practising block phase technique. In this way, future research might build on the results of this study in studying the (optimal) block phase technique for a competitive dive start.

Tor et al. [24,28] reported that on average 56% of the start time is spent underwater and that the underwater phase contributes greatly to overall start performance. The current findings are consistent with these previous findings because, as explained, the entry state defines the initial conditions of the underwater phase and thus affects how the underwater phase unfolds. In addition, the current study found a substantial increase in variation (from 0.02 to 0.38) between the time from take-off to the 5-m line and the time from take-off to the 15-m line, which may well be due to differences in the glide phase and underwater kicking ability [42]. Despite this increased variation, the same entry state variables were found for start performance related to the 5-m and 15-m line, which underscores the importance of entry state for start performance and the robustness of our findings for different distances and times. Moreover, the difference in relative importance of the variables between the 5-m and 15-m prediction models (Eqs 1 and 2) indicates that the effects of entry state persist after passing the 5-m line. This can be understood by looking at the nature of the variables. E.g., given a certain horizontal COM velocity at entry state, a larger entry distance will cause a higher average horizontal COM velocity until the velocity is stabilized. Since, at the 5-m line, the velocity is still decreasing [11,43], the effect of a larger entry distance will persist after passing this line. In other words, the entry state continues to determine the subsequent phases of the dive start, thus substantiating an essential assumption underlying our approach. This substantiates that the entry state is determinative for all subsequent phases of the dive start. Future research on the dive start is required to detail the precise relation between entry state and underwater phase, and how the underwater phase may be optimised through optimising the entry state.

The main finding of the present study is that start performance is determined to a considerable degree by the collection of kinematic variables defining the swimmer’s entry state. Since entry state is the result of the block phase and the subsequent flight phase, these phases should be focused upon in training activities aimed at improving start performance. Which heuristic guidelines for improving start performance may be gleaned from the present results? In answering this question, it is important to note that horizontal velocity and entry distance contributed about equally well to start performance. Both these variables depend on the magnitude and direction of the velocity vector at take-off, but, given a certain magnitude, require different directions for their optimisation. To maximise the horizontal velocity, it is expected that the velocity vector has to be directed horizontally, whereas to maximise the entry distance, the velocity vector has to be directed around 30 degrees upward as can be derived from the physical laws governing projectile motion. Hence, a trade-off exists in optimising these two entry variables that is mediated by the direction of the velocity vector. It follows from these considerations that, in contrast to the belief of many swim coaches, a higher horizontal velocity does not necessarily lead to a better start performance. A singular emphasis on maximising horizontal velocity in training may well lead to a suboptimal start performance. Coachers and swimmers with them need to search for the optimal combination of horizontal velocity and entry distance, given a certain magnitude of the velocity vector. Of course, training activities may also be aimed at increasing the magnitude of the velocity vector itself, but the possibilities for doing so might be limited due to biomechanical and neuromuscular constraints.

Although this study provided useful outcomes with regard to the relation between entry state and start performance, some limitations should be noted. First of all, not all swimmers included in the study, although highly trained and competitive, were elite swimmers. Therefore, caution should be exercised in generalising the present results to the training of elite swimmers, even though their start performance was found to be similar to that of elite swimmers. In addition, results may not be directly applicable to breaststroke swimmers, since their underwater phase differs from the one in the current study. Secondly, the kinematical analysis was done manually on the basis of video recordings at a frame rate of 50 Hz. Both the manual analysis and the frame rate may have caused inaccuracies in determining the exact location of water entry and the exact position of the body landmarks. However, a test-retest protocol revealed that the maximal error for determining water entry was only 0.01 s, resulting in an estimated maximal horizontal COM position error in the order of 0.04 m. Even though it has been shown [32] that a frame rate of 100 Hz results in smaller errors, the statistical analysis of the current dataset yielded robust results. In part, this may be because it was not necessary to calculate second order derivatives of position data. In addition, erroneous marker positions were corrected using the constrained optimization algorithm of Faber et al. [33]. Thirdly, although the participants’ freshness was monitored and revealed no significant differences, the warm-up protocol was not standardized between sessions, and thus may have led to individual variation in warm-up degree. In future research, participants might be asked to perform a standard start at the start of each session, as the basis for determination of day-to-day variation.

Finally, the statistical analyses conducted focused on linear correlations, even though the relation between entry state and start time may be nonlinear.

## Conclusions

In conclusion, start performance (TTO15) is predicted to a considerable extent by entry state in well-trained, (sub-)elite swimmers. In particular, a better start performance is related to a larger distance from the block at water entry, a higher COM horizontal velocity, and a flatter entry angle at the instant the COM crosses the water line. Distance and COM horizontal velocity at entry were stronger contributors to TTO15 than entry angle. After further validation, the model capturing this relation may provide a basis for studies aimed at identifying optimal block phase kinetics, as well for formulating guidelines for improving the dive start through training.

## Supporting information

S1 Table Table with start and entry variables of all starts included in the study. (XLSX)

S2 Table Squat jump heights from the squat jump test. (XLSX)

S3 Table Points extracted from video-images for analysis of water entry state. (XLSX)

S4 Table Data corresponding to Fig 2. (XLSX)

S1 Appendix Appendix A.
(DOCX)

S2 Appendix Appendix B.
(DOCX)
