# Supplementary material for: Predicting dive start performance from kinematic variables at water entry in (sub-)elite swimmers
Source: PLoS One. 2020 Oct 30;15(10):e0241345. doi: 10.1371/journal.pone.0241345 (PMC7598512; doi:10.1371/journal.pone.0241345)
Supplement: S1 Appendix — Appendix A. (DOCX) [file pone.0241345.s005.docx]

**Appendix A.**

Instructions for the different start conditions

Before every session the following instructions were given (translated from Dutch):

‘You will do eight freestyle starts per session. All starts are performed at maximal effort including the breakout and front crawl swimming until your head reaches the marker at 18m. After the starts, I will tell you if the start was good or if you have to do it again. A start is approved if the requirements for the condition are satisfied, if you start the movement after the start signal, if you indicate that the performance was maximal and if you kept on swimming until the marker was reached.’

‘Today the two conditions are … and … .’ Following by the explanation of the conditions of that session. The instructions and assessment criteria per condition are listed in Table 7. If the swimmer did not meet the assessment criteria, the start had to be repeated.

**Table 7. Instructions and assessment criteria listed per start condition. In addition, the aim, i.e. what we intent to alter with this condition, is explained for each condition.**

| Condition | Instruction | Assessment criteria | Aim |
| --- | --- | --- | --- |
| a. Regular | ‘Start like you would do during an important race.’ | - | - |
| b. Block phase at submaximal effort | ‘You will perform at 50% on the block. Imagine you do just an easy training start to get into the water. However, from the moment you touch the water you will perform at maximal effort.’ | Assessed by the swimmer (was requested by researcher, i.e. ‘Did you really perform at 50% on the block?’). | Decrease magnitude of the velocity vector at take-off |
| c. Steep take-off | ‘Make sure your take-off angle, the angle between your toes and hips at take-off, is very steep. Imagine you jump diagonally up. Try to jump over the backstroke flags.’ | Take-off angle > 5 degrees steeper than average take-off angle of regular starts (assessed by video analysis). | Increase entry distance, decrease horizontal velocity and possibly alter entry angle |
| d. Flat take-off | ‘Make sure your take-off angle, the angle between your toes and hips at take-off, is very flat. Imagine you dive diagonally down.’ | Take-off angle > 5 degrees flatter than average take-off angle of regular starts (assessed by video analysis). | Decrease entry distance and possibly alter entry angle |
| e. Without arms | ‘Just do a normal start, but you may not touch the starting block with your hands.’ | Arms should not touch the block (assessed by visual inspection). | Disrupt regular start |
| f. Block time as short as possible | ‘During this condition, you will try to leave the block as fast as possible. You could do that by shifting your weight to the front and by focussing on the start signal.’ | Block time < 0.03s shorter than average block time of the regular starts (assessed by video analysis). | Disrupt regular start |

Letters a-f are in agreement with the representations in Fig 1.
