# Supplementary material for: Predicting dive start performance from kinematic variables at water entry in (sub-)elite swimmers
Source: PLoS One. 2020 Oct 30;15(10):e0241345. doi: 10.1371/journal.pone.0241345 (PMC7598512; doi:10.1371/journal.pone.0241345)
Supplement: S2 Appendix — Appendix B. (DOCX) [file pone.0241345.s006.docx]

**Appendix B.**

Additional results and statistical checks corresponding to Equation 2

The linear mixed effects analysis to describe the relation between time from take-off to the 5-m line (TTO5) and water entry variables resulted in Eq. 2. The likelihood ratio statistic that was used for comparing the model without water entry variables with the final model (Eq. 2) revealed that start performance was significantly affected by entry state (χ2 (3) = 267, *p* < 0.001). The standard deviation of the residual decreased from 0.06 to 0.04 s when entry state variables were added. According to the resulting model equation, TTO5 was reduced by -0.11 ± 0.01 s (*p* < 0.001, r^2^ = 0.19) for each m covered by entry distance (X_ES_), by -0.14 ± 0.01s (*p* < 0.001, r^2^ = 0.32) for each m/s in COM horizontal velocity at water entry, and by -0.18 ± 0.03s (*p* = 0.001, r^2^ = 0.08) for each radian in entry angle (EA) at COMcrossWL. The variance of TTO5 was 0.02 of which 91.3% was explained by the model, with a near-zero difference between the mean predicted TTO5 and the mean measured TTO5. The likelihood ratio statistic revealed a small significant difference in the goodness-of-fit between the full model and the final model (χ^2^(2) = 33, *p* < 0.001).

Statistical checks of the model’s assumptions revealed linear partial relations and showed variance inflation factors of 1.10 for entry distance, 1.21 for COM horizontal velocity, and 1.22 for entry angle at COMcrossWL. Hence, no collinearity was observed. The Shapiro-Wilk normality test revealed a normal distribution of the random effects (W = 0.92, *p* = 0.19).
